# Coding Client Language in Motivational Interviewing for HIV Medication Adherence Using Self-Determination Theory

**DOI:** 10.1007/s12529-018-09766-z

**Published:** 2019-01-23

**Authors:** Ailbhe Hogan, Delwyn Catley, Kathy Goggin, Michael Evangeli

**Affiliations:** 1 Department of Psychology, Royal Holloway, University of London, Egham, Surrey, TW20 0EX UK; 2 Center for Children’s Healthy Lifestyles and Nutrition, Children’s Mercy Kansas City, Kansas City, USA; 3 Department of Pediatrics, University of Missouri – Kansas City, Kansas City, USA; 4 Health Services and Outcomes Research, Children’s Mercy Kansas City, Kansas City, USA; 5 Schools of Medicine and Pharmacy, University of Missouri − Kansas City, Kansas City, USA

**Keywords:** HIV, Adherence, Motivational interviewing, Self-determination theory

## Abstract

**Background:**

Both motivational interviewing (MI) and self-determination theory (SDT) emphasise the importance of an individual’s autonomy. SDT proposes that motivation is on a continuum with autonomous motivation (AM) at the self-determined end of the spectrum. Whether client speech reflects AM is not coded in MI process studies, however, as it is subsumed under the broader category of change talk (CT). We aimed to code naturalistic speech within MI sessions for HIV medication (antiretroviral) adherence according to whether expressed motivation was autonomous or controlled. We then assessed relationships between adherence and both autonomous/controlled motivational speech (AM/CM) and CT.

**Methods:**

We developed a new coding tool (the SDT coding system: SDTCS) to measure naturally occurring AM speech and CM speech expressed during an MI session targeting antiretroviral (ART) adherence with 62 adults living with HIV (16 female; mean age 40 years). We coded the same sessions using the motivational interviewing skills code (MISC) 2.5 and then examined relationships with on-time ART adherence.

**Results:**

The SDTCS was developed using a combined inductive and deductive approach. Adequate reliability estimates were achieved and the measure possessed good content validity. Naturally occurring AM speech had a stronger relationship to ART adherence in the week after the MI session than CM speech. There was also some evidence that the relationship between AM speech and adherence was stronger than between CT and adherence.

**Conclusion:**

Future refinement and extension of the SDTCS could allow for a more nuanced understanding of client motivational speech that is currently provided by existing coding tools.

## Introduction

Motivational interviewing (MI) is a goal-focused, person-centred counselling style that aims to elicit and strengthen the client’s own motivation for behaviour change [1]. Understanding the active ingredients of MI will allow for appropriate adaptations of this intervention and may improve clinical outcomes, training, practice, and research [2].

Psycholinguistic analyses of MI sessions form the basis of MI process research studies, with MI theorists highlighting the importance of the language clients use about behaviour change [3, 4]. Change talk (CT) is thought to be a key treatment mediator and is associated with behaviour change across diverse populations and behaviours [5, 6]. CT encompasses language describing the desire, ability, reasons, needs, and commitment to change. However, a recent meta-analysis failed to find a relationship between CT and outcome [7]. The absence of an effect at an aggregate level warrants further detailed examination of CT.

Self-determination theory [8] is proposed as a theoretical lens to understand how MI works [9]. SDT proposes that motivation is on a continuum from fully self-determined to non-self-determined action. The more self-determined a behaviour, the more likely a person is to initiate and persist with it. Autonomous motivation (AM) lies at the self-determined end of the spectrum where the behaviour has been chosen. Controlled motivation (CM) refers to engaging in behaviours for externally referenced reasons such as perceived coercion by others, to gain rewards or approval from others or to avoid feelings of guilt or increase feelings of self-worth. AM has been linked to engagement with and completion of alcohol treatment [10], tobacco abstinence [11], and adherence to medication [12], including HIV antiretroviral (ART) medication [13].

Change talk coding tools such as the motivational interviewing skills code [MISC] 2.5 [14] classify CT statements into separate categories (e.g., reasons or commitment for making a change) that do not distinguish the degree of self-determination. This may obscure an important dimension of change talk critical to its impact on behaviour change. To date, there has been no consideration of whether CT reflects apparent AM or CM. A method of classifying CT as likely reflecting AM or CM may enhance its predictive value.

AM and CM are usually measured through self-report questionnaires such as the Treatment Self-Regulation Questionnaire (TSRQ) [15]. While these measures are quick to administer, they possess limitations for examining psychotherapy mechanisms of change. For example, self-report measures are often developed deductively and refined using classical test theory, with data not fitting pre-existing theory discarded. This may limit their content validity [16]. In addition, and most importantly for MI process research, they are not useful in the analysis of client speech during sessions.

Given these limitations, and the varied evidence linking CT and behaviour change, we aimed to develop a novel method of measuring AM speech and CM speech from client speech in MI sessions from an ART adherence trial. We coded *all* speech within a session corresponding to these categories. We predicted that higher levels of ART adherence would be more closely associated with AM than CM speech. We also explored whether AM speech was more closely related to ART adherence than the broader, traditional MI category of CT. This reflects the fact that MI emphasises the effect of the amount, intensity, and sequence of change talk, whereas SDT focuses on the *quality* of change talk [17].

## Methods

This was a secondary analysis of de-identified data collected as part of Project MOTIV8, an RCT exploring the use of MI to increase ART adherence [18]. Ethical approval for the study was first obtained from Institutional Review Boards in 2004.

### MOTIV8 Participants and MI Sessions

Participants were recruited from six government-funded primary care outpatient clinics in Kansas City. Eligible participants were HIV positive, over the age of 18, English-speaking, and taking ART medication. Demographic and clinical information are presented in Table 1.

**Table 1:** Participant demographic and clinical information (*n* = 62)

	All participants	
Variable	Mean (SD)	Number (%)
Age, years	40.1 (10.1)	
Male gender at birth		46 (74.2)
Ethnicity		
African-American		33 (53.2)
White		23 (37.1)
Mixed		5 (8.1)
Other		1 (1.6)
Education		
High School Degree or Less		31 (50.0)
More than High School Degree		31 (50.0)
Sexual orientation		
Heterosexual		27 (43.5)
Homosexual		26 (41.9)
Bisexual		6 (9.7)
Other		1 (1.6)
Choose not to answer		2 (3.2)
Employment		
Full-time		9 (14.5)
Part-time		6 (9.7)
Not currently employed		47 (75.8)
Depressive symptoms^1^		
Above clinical threshold		36 (58.1)
First time taking ART		18 (29.0)
Viral load (copies/ml)—baseline (*n* = 53)		
< 400		53 (100)
≥ 400		0 (0)
CD4 count (cells/mm^3^)—baseline	264.1 (177.7)	

^1^Center for epidemiological studies depression scale (Radloff, 1977)

Individual face to face MI sessions were scheduled at baseline, weeks 1, 2, 6, 11, and 23. Participants also received four telephone sessions (weeks 4, 9, 15, and 19). Sessions lasted, on average, 25 min. The baseline session consisted of information on adherence. Subsequent sessions used one of 11 skills-building modules (e.g., self-monitoring, goal setting). MI for motivation enhancement was the focus of the week 1 therapy session. MI sessions were provided by Master’s degree level professionals, trained and supervised weekly by an expert MI trainer (second author).

### Sample Selection

One hundred thirty-nine participants were randomised to receive the MI intervention in the MOTIV8 RCT. This study focused on the week 1 therapy session (MI for motivation enhancement) as this was delivered to all participants receiving MI. Due to the ceiling effect noted in the RCT data whereby participants reported high motivation to adhere to ART, this study focused on those participants with lower baseline adherence motivation (mean motivation to adhere < 10, *n* = 65). Ten participants with high baseline motivation to adhere (mean motivation to adhere = 10) were randomly selected to achieve a sample of 75 sessions. Of this identified sample, two audio files were missing and in one session, the client spoke in both Spanish and English throughout. Therefore, these three participants were excluded. Of the 72 remaining sessions, a convenience sample of 66 sessions was coded and deemed sufficient for the purposes of the study*.* During data analysis, it was found that four participants had missing adherence data and they were excluded from the study leaving a final sample of *n* = 62. Twelve additional sessions were randomly selected for development of the SDT coding system from the sessions not selected for the main analysis. All transcripts were parsed and coded by the first author.

### Measures

#### ART Adherence

ART adherence data was collected using an electronic pill-cap (Medication Events Monitoring System [MEMS] cap), which captured the date and time when a medication bottle was opened. ART adherence was calculated as the percentage of prescribed ART doses taken on time (within 2 h either side of the scheduled dose time). Data were calculated at three separate intervals: (1) week 1 (the 7-day period before the first MI session); (2) week 2 (the 7-day period after the first MI session); and week 12 (30 days of adherence data prior to the 12th week of the trial).

#### Motivation to Adhere

A brief self-report measure capturing baseline ART adherence motivation was devised. Participants rated from 0 = *not at all* to 10 = *extremely*, their need, reasons, readiness, and commitment to adhere strictly to the ART schedule (*α* = 0.83).

#### Motivational Interviewing Skills Code (MISC) 2.5

Therapy sessions were coded using the MISC 2.5 [14] with twelve sessions (19%) randomly selected for double coding by an undergraduate researcher. Training followed the procedures outlined in Moyers and Martin [19]. The first author spent 30 h of professional training in the use of the MISC 2.5 while an undergraduate researcher spent 15 h in training. The session was parsed into separate speech utterances and a behavioural code was assigned to each one. CT categories are as follows: desire (e.g., “I want to take medication every day”), ability (e.g., “I can stop missing doses”), reason (e.g., “Life will be better if I take my medication”), need (e.g., “I need to stop skipping my medication”), taking steps (e.g., “Last week I set an alarm to remind myself to take the meds”), commitment (e.g., “I’m going to start taking the medication tomorrow”), and other (i.e., change talk not captured by the other categories). To prevent coding drift, weekly coding meetings between the first author and the undergraduate researcher were held after every three sessions. The final author resolved any outstanding disagreements. Cohen’s kappa [20] tested CT inter-rater agreement. There was substantial agreement between raters (kappa = 0.69).

#### SDT Coding System (SDT Coding System: SDTCS)

A coding system was developed to quantify naturally occurring AM and CM speech. Eight of the transcripts selected for coding development were reviewed to identify all references to clients’ motivation to adhere to ART. These adherence reasons were categorised as being either AM speech (e.g., “Taking my ART is important to me”, “I take my meds cause it’s just who I am.”, “I take medication as I value the benefits I get from it”) or CM speech (e.g., “My family/friends/doctor make me take it”, “It makes my loved ones happy when I take my medication”, “I feel guilty if I don’t comply with my medication regime”) and were used as examples in the coding manual. Additional examples were generated following a review of the literature and existing self-report measures of AM and CM. Feedback was sought from one female and one male HIV service user recruited from the UK Children’s HIV Association (CHIVA), a charity for children and young adults living with HIV. These service users were over the age of 18, living with HIV and currently taking ART. The manual was piloted on the four remaining transcripts selected for coding development. Codes were agreed between the first author (a doctoral clinical psychology student) and final author (a qualified clinical psychologist) to produce gold-standard transcripts for training. The manual was further refined and extended based on coding meetings following this piloting process. The final SDTCS manual required the coding of all parsed client speech (parsed using the MISC 2.5) [14] as either AM speech, CM speech, or non-motivational client speech. All client speech which could not be coded as AM or CM speech was coded as non-motivational client speech and accounted for the majority of client speech. This category included what SDT would term “amotivational speech”, which refers to the absence of any intention and thus motivation to engage in behaviour. Coding a statement as a “reason” to engage in a behaviour in the MISC 2.5 is time-specific. Therefore, only statements that related to current or very recent motivation to adhere were coded as AM or CM speech. All references to historical motivation were coded as non-motivational client speech.

#### Coding Training

An undergraduate researcher (different individual to second coder for MISC coding) received 10 h of training in the SDTCS. This involved reading key SDT literature, one-to-one teaching on using the coding manual, and practice coding using the gold-standard transcripts.

#### Reliability-Testing

Fifteen sessions (24%) were randomly selected for double coding by the first author and undergraduate researcher. To prevent coding drift, weekly coding meetings were held after every five sessions coded. Consultation was provided by the final author for any outstanding disagreements.

### Data Analyses

Analysis used SPSS 21. As one code (non-motivational client speech) accounted for the majority of client speech (87%) inter-coder agreement for the SDTCS was assessed using Gwet’s [21] AC1 statistic which has been developed to overcome this prevalence problem [22]. Pearson’s correlations tested the relationships between ART adherence before (week 1) and after the MI session (weeks 2 and 12) and (a) AM speech and CM speech (b) CT. Bootstrapped confidence intervals were used due to skewed data. Follow-up tests for dependent correlations investigated if the correlations between AM speech and adherence, and CM speech and adherence, were significantly different from one another. The correlation coefficients were converted to *z* scores using Fischer’s transformation and established equations were applied to compute the asymptotic covariance of the estimates. Then an asymptotic *z* test was performed [23].

## Results

The overall inter-rater reliability of the SDTCS between the first author and undergraduate researcher, using Gwet’s AC1 statistic, was 0.89 (near perfect agreement). Gwet’s AC1 statistic of 0.95 (perfect agreement) was achieved for non-motivational client speech, 0.45 (moderate agreement) for CM speech and 0.32 (fair agreement) for AM speech. Due to the lower reliabilities for AM speech and CM speech, all categorizations were verified by the final author before a final coding decision was made.

Table 2 displays the descriptive statistics for each variable.

**Table 2:** Descriptive statistics of coding and adherence data

Variable	Median	Interquartile range	Mean	SD
Week 1 (% doses taken on time)	85.71	62.50–100.00	76.05	29.85
Week 2 (% doses taken on time)	89.29	57.14–100.00	74.25	33.49
Week 12 (% doses taken on time)	78.57	53.00–95.21	69.42	29.38
Autonomous motivation speech (count)	3.00	1.75–6.00	4.26	3.94
Controlled motivation speech (count)	6.50	4.00–10.00	8.48	8.55
Change talk (count)	38.00	30.75–55.75	45.87	24.69

Across the three time points for ART adherence data the correlations were larger for AM speech than for CT, which in turn were larger than for CM speech (see Table 3).

**Table 3:** Pearson’s correlation coefficients (*r*) between ART adherence (weeks 1, 2, 12) and autonomous and controlled motivation speech and change talk (*n* = 57)

Variable	Autonomous motivation speech	Controlled motivation speech	Change talk
Week 1 (% of doses taken on time)	0.09 [− 0.12, 0.26]	− 0.01 [− 0.29, 0.35]	0.01 [− 0.27, 0.32]
Week 2 (% of doses taken on time)	0.28* [0.08, 0.44]	− 0.07 [− 0.34, 0.27]	0.03 [− 0.21, 0.30]
Week 12 (% of doses taken on time)	0.19 [− 0.04, 0.38]	0.03 [− 0.21, 0.20]	0.09 [− 0.09, 0.30]

*Note:* BCa 95% confidence intervals in parentheses below each *r* value

*p < 0.05

The relationships between CM speech and ART adherence were not significant at any of the time points. The relationships between AM speech and ART adherence at weeks 1 and 12 were not significant but there was a medium effect sized significant positive correlation between AM speech and ART adherence in the week following the MI session (week 2), *r* (60) = 0.28, *p* < 0.05, 95% BCa CI [0.06, 0.45].

The association between AM speech and ART adherence after the MI session (week 2) was significantly larger than between CM speech and week 2 ART adherence; *z* = 2.37, two-tailed, *p* = 0.02. The association between AM speech and ART adherence was not significantly larger than the association between CM speech and ART adherence at either week 1 (*z* = 0.51, two-tailed, *p* = 0.61) or week 12 (*z* = 1.03, two-tailed, *p* = 0.30).

## Discussion

We aimed to (a) quantify AM speech and CM speech, as well as CT, through coding client speech during MI sessions and (b) investigate their relationships with ART adherence. This study is the first to attempt to measure within-session AM and CM speech. We were able to develop a coding scheme that achieved good inter-rater agreement across categories and fair to moderate agreement for SDT-specific categories. The reliability estimates at the subscale level are in line with a reliability study of the MISC which found that 43% of the subscales achieved poor reliability estimates (− 0.03–0.36) and these were mainly low-frequency categories [24]. Our study represents a promising first attempt at testing SDT’s theory of how motivation is associated with behaviour during the therapeutic process.

We predicted that AM speech would be more closely related to ART adherence than CM speech. The results offer tentative support for this prediction as there was a significant medium effect sized relationship between AM speech and ART adherence in the week following the MI session (week 2) and there was a non-significant positive association between ART adherence at the same time point and CM speech (with the former correlation being significantly larger than the latter). These results are in line with research showing that self-reported AM is related to successful medication adherence [12, 13] and with research showing that self-reported AM is better than CM at predicting behaviour change [10].

It is also promising that a medium effect size was detected in the relationship between AM speech and ART adherence given that a previous study using the TSRQ was only able to find a small effect (*r* = 0.15) between AM and ART adherence [13]. Although we found no significant relationship between either AM or CM speech and ART adherence at week 1 (before the coded MI session) and week 12, these findings likely reflect AM speech levels changing as a result of the MI session and the effects of these changes on adherence weakening over time.

We also examined the association between CT and adherence and found that the magnitude of the relationships between AM speech and adherence appeared to be greater (although the non-independence of AM speech and CT meant that it was not possible to test this statistically). This suggests that more attention to the theoretical basis of the relationship between change talk and behaviour change could improve understanding of the role of change talk in MI. The SDTCS offers a potentially useful tool for enhancing the MISC to incorporate SDT principles.

One area that this paper does not focus on is client language away from change or “sustain talk” (ST). Recent research has highlighted the importance of ST, and a composite variable of CT and ST has been shown to be associated with positive behaviour outcomes [7]. ST does not map directly onto the motivation continuum since AM and CM in SDT refer to different reasons to engage in a behaviour (as expressed in CT) rather than reasons *not to* engage in a behaviour (as expressed in ST). Also, amotivation—as understood from an SDT perspective—is lacking the intention to act/acting without intent while ST refers to a desire to continue with an existing way of acting rather than making a change. Future research might focus on resolving how the concept of ST fits within the SDT theoretical framework. It might be important to consider how autonomous or controlled reasons not to engage in a behaviour or to maintain the status quo might be related to outcomes.

Although the SDTCS appears promising, further research is needed to establish its reliability and refine criteria. For example, it would be helpful to develop criteria that could further enhance inter-rater agreement alongside further refinement of the coding tool, a longer coding training requirement, and closer exploration of statements where there is a lower level of agreement.

Another potential limitation is the tool development involved the use of MI sessions with participants who rated themselves as highly motivated to change however this was deemed necessary to develop varied examples of AM and CM reasons for medication adherence. Further, it would be helpful to code AM speech and CM speech across a broader range of target behaviours. This may allow for a more nuanced understanding of client language that is currently provided by existing MI process tools (relying on the broader categories of CT) which may further add to the understanding of how MI works. Despite these limitations, this preliminary evaluation of the SDTCS suggests that it may be a useful tool for studying the process of MI and evaluating the effectiveness of MI through the lens of SDT.
